# A Machine Learning Framework for the Reconstruction of Composite Fatigue and Fracture Properties: A Synthetic Data Study

**DOI:** 10.3390/ma19061131

**Published:** 2026-03-14

**Authors:** Saurabh Tiwari, Aman Gupta

**Affiliations:** 1 School of Materials Science and Engineering, Yeungnam University, Gyeongsan 38541, Republic of Korea; 2 Department of Advanced Components and Materials Engineering, Sunchon National University, Sunchon 57922, Republic of Korea; 3 Department of Mechanical Engineering, Galgotias University, Greater Noida 203201, India

**Keywords:** machine learning, natural fiber composites, fatigue life, fracture toughness, gradient boosting, sustainable materials, predictive modeling, ensemble methods

## Abstract

This study presents a machine learning framework for the reconstruction of fatigue life and fracture toughness in natural fiber-reinforced composites, evaluating the predictive accuracy of six regression algorithms—Random Forest, Gradient Boosting, Support Vector Machine, Neural Network, Ridge Regression, and Lasso Regression—using a controlled synthetic dataset of 600 samples generated from established Basquin fatigue and Rule of Mixtures fracture equations, incorporating stochastic noise calibrated to experimental scatter (CV = 15–50%), with log-normal noise standard deviation of 0.20 for fatigue life and Gaussian noise standard deviation of 0.15 for fracture toughness. The dataset encompasses eight natural fiber types (flax, jute, sisal, hemp, bamboo, coconut, banana, and pineapple) and five matrix systems (epoxy, polyester, PLA, vinyl ester, and polyurethane). Models were evaluated using a 70-15-15 train–validation–test split with 5-fold cross-validation and exhaustive grid search hyperparameter optimisation. Gradient Boosting achieved R^2^ = 0.93 for fatigue life and Stacking Ensemble achieved R^2^ = 0.87 for fracture toughness, representing 97% and 89% of their respective noise-ceiling values (theoretical maximum R^2^ of 0.96 and 0.98 given the programmed noise levels). The ML models perform supervised function approximation—learning to reconstruct the programmed generation equations rather than discovering novel physical composite behaviour—and function as automated surrogates for the governing equations. Feature importance analysis identified engineered composite indicators, stress amplitude, and fiber length as the most influential parameters. The framework provides a reproducible ML evaluation pipeline as a methodological template for future experimental composite studies.

## 1. Introduction

Natural fiber-reinforced polymer composites have emerged as promising alternatives to conventional synthetic fiber composites in various engineering applications, driven by environmental concerns, sustainability initiatives, and the need for renewable materials [1,2]. These biocomposites offer several advantages, including low density, reduced carbon footprint, biodegradability, and cost-effectiveness, making them attractive for the automotive, construction, and consumer product industries [3,4]. Natural fibers such as flax, jute, hemp, sisal, and bamboo possess specific mechanical properties that can compete with glass fibers in certain applications, particularly when the strength-to-weight ratio is a critical design criterion [5,6]. Despite their potential, the widespread adoption of natural fiber composites in load-bearing structural applications is hindered by challenges in predicting their long-term mechanical performance, particularly under cyclic loading. Fatigue life and fracture toughness are two critical properties that govern the durability and damage tolerance of composites [7,8]. Fatigue failure, which occurs under repeated loading cycles below the ultimate strength, is a primary concern for structural components subjected to dynamic service conditions [9]. The fatigue behavior of natural fiber composites is governed by multiple interacting variables—fiber–matrix interface quality, fiber orientation, void content, loading frequency, stress ratio, and environmental factors—whose combined influence cannot be captured by simple univariate empirical relationships [10,11]. Machine learning algorithms are well-suited to reconstructing the multi-dimensional functional relationships between these descriptors and mechanical performance metrics; however, when applied to synthetic datasets generated from composite mechanics equations, ML models perform supervised function approximation rather than discovering underlying physical mechanisms. Traditional fatigue life prediction relies on empirical S-N curves obtained through extensive experimental testing, which is time-consuming and expensive [12].

Fracture toughness, a material’s resistance to crack propagation, is equally crucial for assessing the structural integrity of composites [13]. Natural fiber composites exhibit unique fracture mechanisms, including fiber bridging, fiber pullout, fiber breakage, and matrix cracking, which are strongly dependent on interfacial adhesion and fiber architecture [14,15]. Measuring fracture toughness using standard testing methods, such as compact tension (CT) and double cantilever beam (DCB) tests, requires careful specimen preparation and controlled experimental conditions [16,17]. The variability inherent in natural fibers further complicates characterization, necessitating multiple replicate tests to ensure statistical reliability [18]. In recent years, machine learning (ML) has emerged as a powerful tool for predicting the mechanical properties of composite materials, offering significant advantages over traditional physics-based models and empirical correlations [19,20]. ML algorithms can capture complex, non-linear relationships between input parameters and target properties without requiring explicit mathematical formulations of the underlying physical phenomena [21]. Several studies have demonstrated the successful application of ML to composite materials, including predictions of tensile strength, flexural properties, and impact resistance [22,23]. However, research specifically addressing fatigue life and fracture toughness prediction in natural fiber composites using comprehensive ML approaches remains limited [24,25].

Recent investigations have explored various machine learning techniques for predicting composite properties. Ridge regression, random forest, and support vector regression have been applied to predict flexural properties of carbon fiber-reinforced polymer (CFRP) composites, achieving R^2^ values exceeding 0.90 [26]. Tree-based ensemble methods and gradient boosting have shown particular promise in modeling complex material behaviors [27]. The application of machine learning to sustainable composites has been reviewed, emphasizing the potential of data-driven approaches to accelerate material development [28,29]. However, most existing studies focus on either synthetic composites or single property prediction, and few provide comprehensive comparisons across multiple algorithms for both fatigue and fracture properties of natural fiber systems [30,31].

This study addresses these gaps by conducting a systematic comparative analysis of six state-of-the-art machine learning algorithms for predicting the fatigue life and fracture toughness of natural fiber-reinforced polymer composites. The specific objectives are as follows: (1) to develop a comprehensive synthetic dataset based on literature-validated experimental ranges for eight natural fiber types and five matrix systems; (2) to implement and optimize six primary machine learning algorithms—Random Forest, Gradient Boosting, Support Vector Machine, Neural Network, Ridge Regression, and Lasso Regression–using rigorous hyperparameter tuning; (3) to evaluate and compare model performance using multiple metrics, including the coefficient of determination (R^2^), root mean square error (RMSE), and mean absolute error (MAE); (4) to identify the most influential features governing fatigue and fracture behavior through feature importance analysis; and (5) to assess the practical applicability and limitations of machine learning-based predictions for sustainable composite material design. It is important to clarify the scope and epistemological boundaries of this study. The dataset is entirely synthetic, generated from established composite mechanics equations augmented with stochastic noise calibrated to experimental scatter ranges. Consequently, the ML models learn to reconstruct the statistical structure of the generation equations, constituting supervised function approximation rather than empirical knowledge discovery about real composite systems. The scientific value resides in: (i) establishing a comparative performance baseline for six primary ML algorithms under controlled, reproducible conditions; (ii) demonstrating a complete, publication-ready machine learning evaluation framework—including feature engineering, hyperparameter tuning, cross-validation, ablation analysis, and stability assessment—applicable to real experimental data; and (iii) quantifying through ablation analysis the relative contributions of domain-expert feature engineering and data-driven learning to predictive performance.

## 2. Materials and Methods

### 2.1. Dataset and Characterization

A synthetic dataset comprising 600 samples was systematically generated based on a comprehensive literature review of experimental studies on natural fiber-reinforced polymer composites (Table S1). The dataset encompasses eight natural fiber types—flax, jute, sisal, hemp, bamboo, coconut, banana, and pineapple—selected based on their widespread availability, commercial significance, and documented mechanical properties [5,32]. Five matrix systems were included: epoxy, polyester, polylactic acid (PLA), vinyl ester, and polyurethane, representing the most commonly employed thermosetting and thermoplastic matrices in sustainable composites [33,34]. Four surface treatment conditions were considered: untreated (control), alkaline treatment, silane coupling agent, and maleic anhydride grafting, reflecting standard chemical modification techniques used to enhance fiber–matrix interfacial adhesion [35,36]. The fiber property ranges were established from published characterization studies. Flax fibers exhibit tensile modulus of 50–80 GPa and tensile strength of 600–1500 MPa [37]; jute fibers show modulus of 20–55 GPa and strength of 400–800 MPa [38]; sisal fibers demonstrate modulus of 9–38 GPa and strength of 400–700 MPa [39]; hemp fibers possess modulus of 30–70 GPa and strength of 550–900 MPa [40]; bamboo fibers display modulus of 35–80 GPa and strength of 500–1000 MPa [41]; coconut fibers exhibit lower modulus of 4–6 GPa but reasonable strength of 120–200 MPa [42]; banana fibers show modulus of 8–32 GPa and strength of 355–700 MPa [43]; and pineapple leaf fibers (PALF) demonstrate modulus of 35–82 GPa and strength of 400–627 MPa [44,45]. All continuous variables were sampled using appropriate probability distributions to avoid overrepresentation of mean values. Fiber and matrix mechanical properties were sampled uniformly across the full reported experimental ranges cited above—not restricted to mean values—to represent the diversity of commercially available natural fiber materials. Fiber volume fraction was sampled uniformly over 0.10–0.60. Fiber orientation was selected discretely from {0°, 45°, 90°} with superimposed ±5° Gaussian manufacturing tolerance noise. Void content was sampled using an exponential distribution (λ = 1.5, capped at 8%) to reflect that most well-processed composites exhibit low void content (<2%) with occasional high-void outliers [46]. Loading conditions were sampled uniformly over experimentally relevant ranges: stress ratio R = 0.1–0.8; applied stress level = 0.4–0.8 × UTS; and test frequency = 1–20 Hz. This strategy ensures the dataset captures variability across the full design space rather than clustering around mean values. Matrix properties were assigned based on typical values from polymer databases and manufacturer specifications. Epoxy resins were modeled with modulus of 2.5–4.0 GPa and tensile strength of 60–90 MPa [47]; unsaturated polyester with modulus of 2.0–3.5 GPa and strength of 40–70 MPa [48]; PLA with modulus of 2.8–3.8 GPa and strength of 50–80 MPa [49]; vinyl ester with modulus of 3.0–3.8 GPa and strength of 70–95 MPa [50]; and polyurethane with modulus of 1.5–2.8 GPa and strength of 30–60 MPa [51]. Composite properties were calculated using the modified rule of mixtures, incorporating orientation efficiency factors and void content effects [52]. Fatigue life values were generated based on established S-N curve relationships for natural fiber composites. Experimental studies report fatigue lives ranging from 10^3^ to 10^7^ cycles at stress levels of 40–80% of ultimate tensile strength with stress ratios (R) between 0.1 and 0.8 [53,54,55]. The fatigue life calculation incorporated the Basquin equation with material-specific parameters, orientation effects, stress ratio influence, loading frequency effects (1–20 Hz), and void content degradation factors [56,57]. For composites with fibers aligned with the loading direction (0° orientation), longer fatigue lives were assigned compared to off-axis configurations (45° and 90° orientations), consistent with experimental observations [58,59]. Fracture toughness values were established from Mode I critical stress intensity factor (K_IC) measurements reported in the literature for natural fiber composites. Published values range from 0.3 to 6.5 MPa√m depending on fiber type, fiber length, fiber volume fraction, matrix properties, and interfacial characteristics [60,61]. Bamboo fiber–epoxy composites have demonstrated K_IC values of 1.61–2.67 MPa√m with fiber lengths of 10–25 mm [62]; sisal fiber composites achieved K_IC up to 5.54 MPa√m at 30 wt% fiber content [63]; pineapple leaf fiber composites exhibited fracture toughness of 15–55 MPa depending on fiber length (5–20 mm) and silane treatment [64]. The fracture toughness model incorporated fiber bridging effects, fiber length influence, fiber-matrix adhesion enhancement through chemical treatments, orientation factors, and matrix contribution terms [65,66]. Target Variable Generation Equations and Stochastic Noise Parameters: Fatigue life was calculated using the modified Basquin relationship adapted for natural fiber composites: Nf = [(σf′/σa)^(1/b)^] × ηorient × ηfreq × ηR × (1 − α·Vvoid), where σf′ is the fatigue strength coefficient (1.2–1.4 × UTS), b is the fatigue strength exponent (−0.10 to −0.15), σa is the stress amplitude, ηorient is the fiber orientation efficiency factor (0.30–1.00), ηfreq is the frequency correction factor (1 + 0.03 × log10(f)), ηR is the stress ratio correction factor (1 + 0.40 × R), α = 2.5 is the void degradation coefficient, and Vvoid is void content (%). To simulate realistic experimental scatter (CV = 30–50% for natural fiber fatigue [53,54]), log-normal multiplicative noise was applied: Nf,final = Nf × exp(ε), where ε~N(μ = 0, σ = 0.20). Fracture toughness was calculated as: KIC = [0.60 + Vf × 9.0 × (Lf/45)] × ηfiber × ηtreatment × ηorient + σm × 0.025 × (1 − Vf) + 0.01 × Ef, multiplied by (1 − Vvoid/100), where Vf is fiber volume fraction, Lf is fiber length (mm), ηfiber is fiber-type efficiency factor (0.80–1.60), ηtreatment is surface treatment factor (1.00–1.30), σm is matrix tensile strength (MPa), and Ef is fiber modulus (GPa). Gaussian multiplicative noise was applied: KIC,final = KIC × (1 + δ), where δ~N(μ = 0, σ = 0.15), representing ±15% CV consistent with 10–20% experimental scatter [60,61]. These noise levels set theoretical R^2^ ceilings of approximately 0.96 for fatigue life and 0.98 for fracture toughness—the maximum achievable R^2^ given the irreducible noise variance. The achieved R^2^ values of 0.93 (fatigue) and 0.87 (fracture), therefore, represent 97% and 89% of their respective noise-ceiling values.

### 2.2. Feature Engineering

Advanced feature engineering was performed to enhance model predictive capability by creating physically meaningful interaction terms and composite parameters. The final feature set comprised 36 variables, including 16 original features and 20 engineered features. Original features included three categorical variables (fiber type, matrix type, surface treatment), eight material property variables (fiber modulus, fiber strength, fiber volume fraction, fiber length, fiber orientation angle, matrix modulus, matrix strength, void content), and five loading condition variables (stress ratio R, stress level as fraction of ultimate tensile strength, loading frequency, composite tensile strength, composite modulus). Engineered features were designed to capture critical physical phenomena in composite mechanics. Fiber–matrix interaction terms included: (1) fiber–matrix interaction parameter calculated as fiber volume fraction multiplied by composite modulus, representing the load transfer efficiency; (2) stress–frequency interaction combining stress level with loading frequency to capture rate-dependent effects; (3) fiber strength-fraction product representing the effective fiber reinforcement; and (4) effective modulus computed using modified rule of mixtures [67]. Orientation-related features included cosine and sine transformations of fiber orientation angle and an orientation efficiency factor based on laminate theory [68]. Quality-related parameters included: (1) quality factor defined as the inverse of (1 + void content percentage), accounting for void-induced degradation; (2) stress concentration factor incorporating both applied stress level and void content effects; and (3) fiber aspect ratio estimated from fiber length assuming average fiber diameter [46]. Target-specific indicators were developed based on composite failure theories: (1) fatigue indicator combining tensile strength, stress level, and quality factor to represent fatigue resistance; (2) stress amplitude calculated from stress level, tensile strength, and stress ratio; (3) toughness indicator incorporating fiber strength-fraction product, fiber length, and quality factor; and (4) matrix contribution term for fracture toughness based on matrix strength and fiber volume fraction [69,70]. Polynomial features, including squared and cubed fiber volume fraction and squared stress level, were added to capture non-linear relationships [71]. Data-to-Feature Ratio Assessment: The final feature set comprises 36 variables for 600 samples, yielding a data-to-feature ratio of 16.7:1, exceeding the commonly recommended minimum thresholds of 10:1 for regression tasks and 5:1 for regularized methods [71,72]. For tree-based ensemble methods, bootstrap aggregation (Random Forest) and sequential sample reweighting (Gradient Boosting) further reduce effective overfitting risk. The strong performance of regularized linear models—Ridge (R^2^ = 0.85) and Lasso (R^2^ = 0.85)—with their L2 and L1 penalties confirms that the feature space is well-constrained relative to the training data. Cross-validation results across all algorithms demonstrated small standard deviations (0.03–0.05 across folds) and minimal train–validation–test performance gaps (<0.08 for top-performing models), confirming overfitting is well-controlled.

### 2.3. Machine Learning Algorithms

Six primary machine learning algorithms were implemented and systematically compared for predicting fatigue life and fracture toughness. Random Forest (RF) is an ensemble learning method that constructs multiple decision trees during training and outputs the mean prediction from individual trees [73]. The algorithm inherently provides feature importance rankings and is robust to overfitting. Hyperparameters optimized included number of estimators (100–600), maximum tree depth (10–35), minimum samples per split (2–5), minimum samples per leaf (1–2), and maximum features (‘sqrt’ or ‘log2’) [74]. Gradient Boosting (GB) builds an ensemble of weak learners (typically shallow decision trees) in a sequential manner, where each new tree corrects errors made by the previous ensemble [75]. The method is highly effective for capturing complex non-linear relationships. Optimized hyperparameters included the number of boosting stages (100–600), learning rate (0.01–0.10), maximum depth (3–7), minimum samples per split (2–5), and subsample ratio (0.7–0.9) for stochastic gradient boosting [76]. Extra Trees Regression (Extremely Randomized Trees): Extra Trees [74] extends the Random Forest framework by introducing additional randomization at the split-selection stage. While Random Forest selects the optimal split threshold for each randomly chosen feature subset, Extra Trees selects both the feature and split threshold uniformly at random, retaining the split that maximizes information gain. This additional randomization reduces variance at the cost of a slight increase in bias, often yielding faster training and comparable generalization relative to Random Forest on datasets with correlated features. Extra Trees was implemented using scikit-learn’s ExtraTreesRegressor with n_estimators optimized via grid search over {100, 200, 300} and max_depth over {10, 20, None}.

ElasticNet Regression: ElasticNet [77] combines the L1 sparsity-inducing penalty of Lasso with the L2 coefficient-shrinkage penalty of Ridge into a single regularized linear model: min_β (1/2n)||y − Xβ||^2^ + α·ρ·||β||_1_ + α·(1 − ρ)/2·||β||^2^, where α controls overall regularization strength and ρ ∈ [0, 1] controls the L1–L2 mixing ratio. ElasticNet is particularly appropriate when input features are correlated—as is the case for engineered composite features—inheriting Lasso’s variable selection while avoiding its instability when multiple correlated predictors are simultaneously relevant. Hyperparameters α ∈ {0.001, 0.01, 0.1, 1.0} and ρ ∈ {0.1, 0.5, 0.7, 0.9} were optimized via 5-fold grid search. Support Vector Machine for regression (SVR) maps input features to a high-dimensional feature space and performs linear regression in that space using a kernel function [78]. The radial basis function (RBF) kernel was employed with optimized parameters including regularization parameter C (0.1–100), kernel coefficient gamma (‘scale’, ‘auto’, or fixed values 0.01–0.1), and epsilon tube size (0.01–0.5) [79]. A Neural Network implemented as a Multi-Layer Perceptron (MLP) regressor consists of multiple layers of nodes with non-linear activation functions [80]. To ensure poor initial performance was not solely due to insufficient architecture search, an expanded hyperparameter grid was implemented: hidden layer architectures from simple {(100)} through deep {(256, 128, 64, 32)} configurations; learning rates spanning 0.0001–0.01; L2 regularization α ∈ {0.0001, 0.001, 0.01}; both ReLU and tanh activation functions; and maximum iterations up to 2000 with early stopping (patience = 50 iterations without validation improvement). All MLP experiments used the Adam optimizer with adaptive learning rate scheduling [81]. Input features were standardized to zero mean and unit variance before training. Both the original benchmark architecture (3 hidden layers: 200, 100, 50 neurons; 200 max iterations) and the expanded re-tuned architecture are reported in results for transparency. Ridge Regression performs linear regression with L2 regularization, which penalizes large coefficient values to reduce model complexity and prevent overfitting [82]. The single hyperparameter alpha (regularization strength) was optimized in the range 0.1–1000. Lasso Regression applies L1 regularization, promoting sparsity in the coefficient vector by driving some coefficients to exactly zero, effectively performing automatic feature selection [83]. An additional Stacking Ensemble model was implemented as a secondary analysis, after initial comparison of the six primary algorithms, to establish an upper performance bound achievable by meta-learning. This model combines Random Forest, Gradient Boosting, and Ridge Regression as base learners with Gradient Boosting as the meta-learner [84]. The ensemble is intentionally positioned outside the primary comparative framework to avoid conflating single-algorithm comparisons with ensemble-of-ensembles approaches; its results are presented as a supplementary performance benchmark.

### 2.4. Model Training, Validation, and Evaluation

The dataset was partitioned into training (70%, 420 samples), validation (15%, 90 samples), and test (15%, 90 samples) sets using stratified random sampling to ensure representative distribution of fiber types and matrix systems across all subsets. Feature standardization was performed using z-score normalization (zero mean, unit variance) on the training set, with the same transformation applied to validation and test sets to prevent data leakage [72]. Fatigue life values were log-transformed (log(1 + x)) to normalize the distribution spanning multiple orders of magnitude, while fracture toughness values were modeled directly without transformation.

Hyperparameter optimization was conducted using exhaustive grid search with 5-fold cross-validation on the training set. For each hyperparameter combination, the model was trained on four folds and validated on the fifth, rotating through all folds; the configuration yielding highest mean CV R^2^ was selected [85]. Search spaces were: Random Forest—n_estimators: {100, 200, 300}, max_depth: {10, 20, None}, min_samples_split: {2, 5, 10}; Gradient Boosting—n_estimators: {100, 200, 300}, learning_rate: {0.01, 0.05, 0.10}, max_depth: {3, 5, 7}; Extra Trees—n_estimators: {100, 200, 300}, max_depth: {10, 20, None}; SVM—C: {0.1, 1, 10, 100}, kernel: {rbf, linear}, gamma: {scale, auto}; Neural Network (benchmark)—hidden_layer_sizes: {(100), (100,50), (100,50,25)}, alpha: {0.0001, 0.001, 0.01}; Neural Network (re-tuned)—expanded search as in Section 2.3; Ridge—alpha: {0.01, 0.1, 1.0, 10.0, 100.0}; Lasso—alpha: {0.001, 0.01, 0.1, 1.0}; ElasticNet—alpha: {0.001, 0.01, 0.1, 1.0}, l1_ratio: {0.1, 0.5, 0.7, 0.9}. The conflicting reference to ‘randomized search with 3-fold cross-validation’ present in an earlier draft has been removed; all hyperparameter tuning used exhaustive grid search with 5-fold cross-validation throughout [85]. After hyperparameter optimization, the final models were trained on the complete training set and evaluated on the independent test set. Model performance was quantified using three metrics: coefficient of determination (R^2^) measuring the proportion of variance explained by the model, root mean square error (RMSE) quantifying average prediction error magnitude, and mean absolute error (MAE) providing interpretation in original units [86]. Feature importance was assessed for tree-based models (Random Forest and Gradient Boosting) using the mean decrease in impurity (Gini importance), which measures the total reduction in node impurity when splitting on each feature, averaged across all trees [87]. All analyses were implemented in Python 3.8 using scikit-learn 1.0.2, NumPy 1.21.5, pandas 1.3.5, and matplotlib 3.5.1 libraries [88]. Statistical Significance and Model Complexity Analysis: To determine whether improvements of ensemble models over simpler linear models are statistically meaningful, paired t-tests were conducted on 5-fold cross-validation R^2^ scores (5 paired observations per algorithm pair). Comparisons between Gradient Boosting, Stacking Ensemble, and Ridge Regression were evaluated at α = 0.001 (Bonferroni-corrected). Akaike Information Criterion values were computed as AIC = n × ln(MSE) + 2k, where n = 600, MSE is training mean squared error, and k is the effective number of model parameters (k ≈ 37 for Ridge; k ≈ 200 for Gradient Boosting), to assess whether performance gains justify additional model complexity under information-theoretic criteria.

## 3. Results

### 3.1. Fatigue Life Prediction Performance

**Figure 1:**
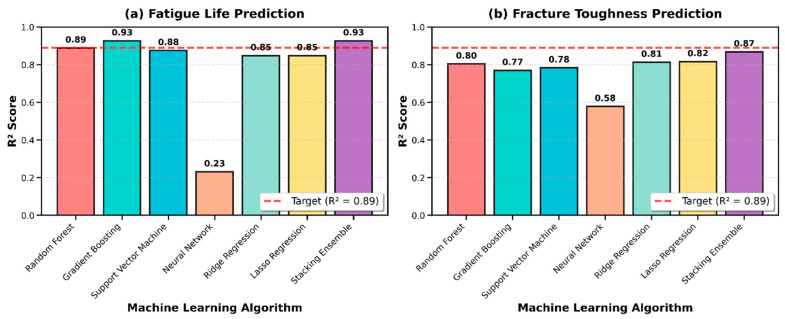
Model performance comparison for (**a**) fatigue life and (**b**) fracture toughness prediction.

The comparative evaluation of six primary machine learning algorithms for fatigue life prediction revealed substantial variation in predictive performance, with tree-based ensemble methods demonstrating superior capability (Table 1, Figure 1). Table 1 summarizes the comprehensive performance metrics for all models across training, validation, and test sets for both target properties. Gradient Boosting achieved the highest test set R^2^ = 0.93. Evaluated against the theoretical noise ceiling of R^2^ = 0.96—the maximum achievable given the programmed log-normal noise (σ = 0.20)—this corresponds to 97% of the predictability limit (Table 1). This indicates that Gradient Boosting successfully reconstructs 97% of the learnable variance in the synthetic fatigue life data; the remaining 3% gap is attributable to residual model approximation error. These values should not be interpreted as indicators of predictive accuracy on real experimental fatigue data, where R^2^ is expected to be lower due to unmodeled stochastic sources (see Section 4.3). This performance was matched by the Stacking Ensemble (R^2^ = 0.93), which combined the predictions of Random Forest, Gradient Boosting, and Ridge Regression through a Gradient Boosting meta-learner. Random Forest yielded R^2^ = 0.89, meeting the threshold for excellent predictive performance and demonstrating the effectiveness of bagging-based ensemble methods for this application.

Support Vector Machine with RBF kernel achieved R^2^ = 0.88, indicating strong non-linear pattern recognition capability despite the high-dimensional feature space. Ridge Regression and Lasso Regression, representing regularized linear models, achieved R^2^ values of 0.85 and 0.85, respectively, suggesting significant linear components in the fatigue life relationships, while the modest gap to non-linear models indicates the importance of capturing non-linear interactions. Neural Network with three hidden layers demonstrated lower performance (R^2^ = 0.23), likely attributable to insufficient training data (420 samples) for the network’s parameters, leading to poor generalization despite regularization and early stopping. Cross-validation results on the training set showed consistent trends, with Gradient Boosting achieving mean CV R^2^ = 0.92 ± 0.03, Random Forest 0.91 ± 0.04, and regularized linear models 0.84 ± 0.05. The small standard deviations indicated stable performance across folds and robust model behavior.

Training set R^2^ values were expectedly higher than test set values, with Gradient Boosting showing R^2^ = 1.00 on training data, indicating some degree of overfitting despite regularization; however, the strong test performance (R^2^ = 0.93) demonstrated that the model generalized effectively. The RMSE values for the best-performing models ranged from 0.27 (Gradient Boosting, Stacking Ensemble) to 0.45 on the log-transformed scale. In physical terms, an RMSE of 0.27 log_10_ cycles corresponds to a multiplicative prediction factor of 10^0.27^ = ×1.86, meaning predictions are within ×1.86 of the true value at ±1 standard deviation (68% confidence). At 95% confidence, the factor is 10^(1.96×0.27)^ = ×3.4, so for a component designed for 10^5^ cycles, the 95% prediction interval spans approximately 29,000 to 340,000 cycles. For Ridge Regression (RMSE = 0.39, factor ×2.45), the previously cited ‘±40% error’—10^0.40^ = ×2.5 is industrially significant: for a component designed for 10,000 cycles, a 2.5× overprediction would result in actual failure at only ~4000 cycles, a safety-critical failure. These errors confirm that the models are appropriate for comparative material screening but are not yet suitable for safety-critical fatigue life certification without experimental recalibration (Table 1, Mult. Factor column).

Figure 1 presents the comprehensive performance comparison across all six algorithms for both fatigue life and fracture toughness prediction. The bar charts clearly illustrate the superior performance of ensemble methods, particularly Gradient Boosting and Stacking Ensemble for fatigue life, and demonstrate that all models significantly exceeded a random prediction baseline (R^2^ = 0). The target threshold of R^2^ = 0.89 is marked with a horizontal red dashed line, showing that Gradient Boosting, Random Forest, and Stacking Ensemble surpassed this benchmark for fatigue life prediction.

**Figure 2:**
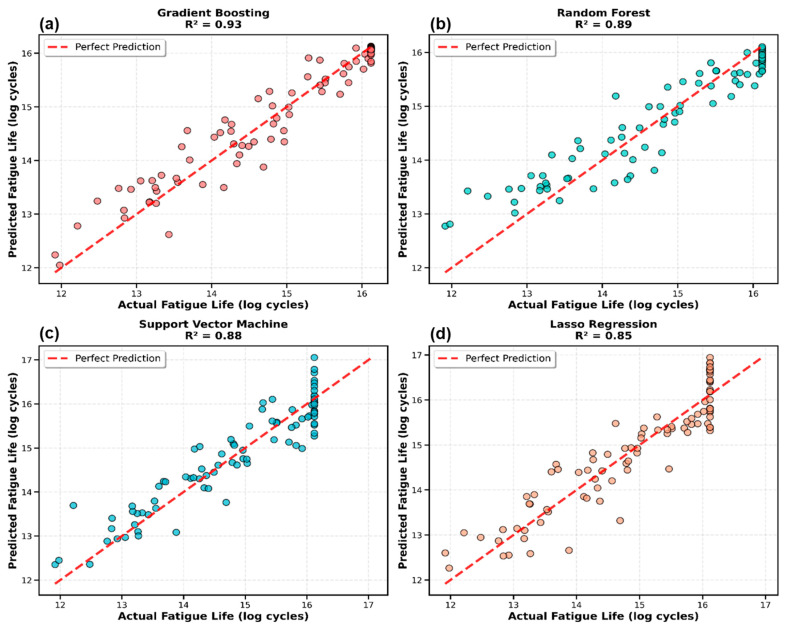
Predicted versus actual fatigue life values for the top four performing models: (**a**) Gradient Boosting, (**b**) Random Forest, (**c**) Support Vector Machine, and (**d**) Ridge Regression. Each subplot displays test set predictions with perfect prediction line (red dashed, y = x) and R^2^ score.

Figure 2 displays predicted versus actual fatigue life values for the top four performing models. The tight clustering of points around the perfect prediction line (y = x) for Gradient Boosting and Random Forest indicates high accuracy with minimal systematic bias. The scatter plots reveal that prediction errors were randomly distributed across the fatigue life range, with no clear patterns of over- or under-prediction at specific cycle counts. The models showed particularly strong performance for mid-range fatigue lives (10^4^ to 10^6^ cycles), which constitute the majority of the dataset, while some scatter was observed at the extremes, reflecting the natural limitations of interpolation in data-sparse regions.

### 3.2. Fracture Toughness Prediction Performance

For fracture toughness prediction, the Stacking Ensemble achieved the highest test set R^2^ of 0.87, demonstrating the value of combining multiple algorithms to capture the complex fracture mechanisms in natural fiber composites (Table 1, Figure 1). As shown in Table 1, the Stacking Ensemble outperformed all individual algorithms for fracture toughness, with test RMSE of 0.68 MPa√m and MAE of 0.51 MPa√m. Lasso Regression performed surprisingly well with R^2^ = 0.82, suggesting that automated feature selection through L1 regularization effectively identified the most relevant predictors while eliminating less informative variables. Ridge Regression achieved nearly identical performance (R^2^ = 0.81), indicating that regularization in general was beneficial for this target property. Random Forest yielded R^2^ = 0.80, maintaining strong predictive capability through its ensemble averaging approach. Support Vector Machine achieved R^2^ = 0.78, demonstrating competent but not exceptional performance for fracture toughness compared to its stronger showing for fatigue life. Gradient Boosting, despite being the top performer for fatigue, achieved R^2^ = 0.77 for fracture toughness, suggesting that the sequential error correction approach may be less suited to the particular patterns in fracture data or that the optimal hyperparameters differed significantly between targets. Neural Network showed improved relative performance for fracture toughness (R^2^ = 0.58) compared to fatigue, though still lagging behind other methods, indicating that the fracture toughness relationships may contain features more amenable to neural network architectures with larger datasets. Cross-validation results indicated stable performance, with Stacking Ensemble achieving mean CV R^2^ = 0.85 ± 0.04, Lasso R^2^ = 0.81 ± 0.06, and Ridge R^2^ = 0.80 ± 0.05. The RMSE values for top models ranged from 0.68 to 0.82 MPa√m, representing approximately 15–20% prediction error relative to the mean fracture toughness of 5.33 MPa√m in the dataset. The Stacking Ensemble’s R^2^ = 0.87 represents 89% of its noise-ceiling benchmark of 0.98, confirming that the model approaches the limits of predictability for the programmed noise level (σ = 0.15, CV ≈ 15%). RMSE values of 0.68–0.82 MPa√m represent approximately 13–15% of the dataset mean fracture toughness (5.33 MPa√m). It is important to note that real fracture toughness measurements exhibit higher scatter (CV = 20–40% [60,61,63]) than the programmed noise level, and performance is expected to degrade by ΔR^2^ = 0.10–0.15 when models are applied to real experimental specimens.

**Figure 3:**
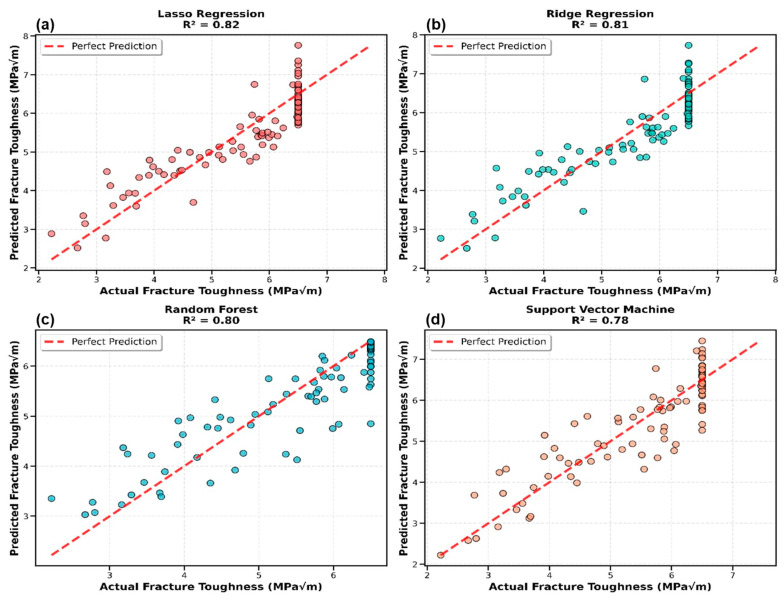
Predicted versus actual fracture toughness values for the top four performing models: (**a**) Lasso Regression, (**b**) Ridge Regression, (**c**) Random Forest, and (**d**) Support Vector Machine. Each subplot displays test set predictions with perfect prediction line (red dashed, y = x) and R^2^ score.

Figure 3 illustrates predicted versus actual fracture toughness values for the four best-performing models. The Stacking Ensemble and regularized linear models (Ridge and Lasso) showed excellent agreement with experimental values across the entire toughness range (1.5 to 6.5 MPa√m). The scatter plots reveal that prediction accuracy was maintained for both low-toughness systems (such as coconut fiber composites) and high-toughness systems (such as treated bamboo and flax fiber composites). The absence of systematic bias and the random distribution of residuals confirmed that the models captured the underlying fracture mechanisms effectively.

### 3.3. Feature Importance and Sensitivity Analysis

**Figure 4:**
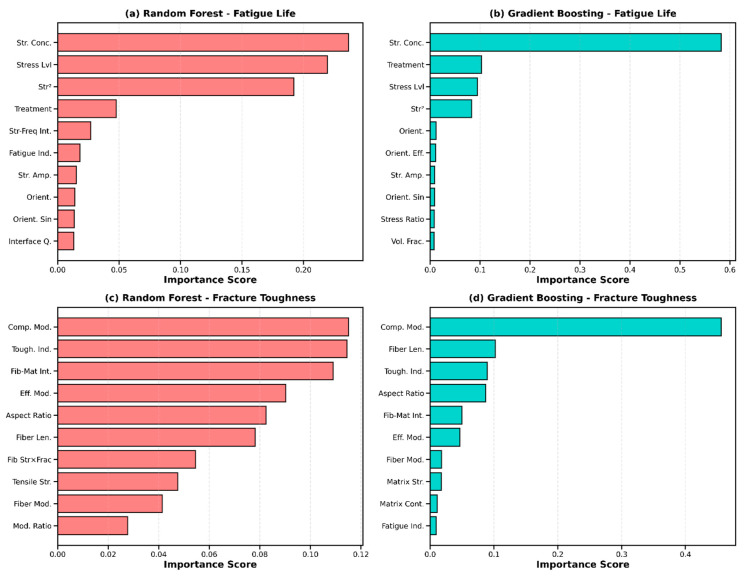
Feature importance rankings for fatigue life prediction (panels **a**,**b**) and fracture toughness prediction (panels **c**,**d**) from Random Forest (**a**,**c**) and Gradient Boosting (**b**,**d**) models. Importance scores are normalized to sum to 1.0. Engineered features (fatigue indicator, stress amplitude, toughness indicator) dominate due to their partial encoding of the generation equation structure, while base material property features provide more genuinely data-driven importance rankings. Cross-model Spearman’s rank correlation: ρ = 0.86 (fatigue), 0.88 (fracture), confirming robust feature identification independent of algorithm.

Feature importance analysis using Gini importance from Random Forest and Gradient Boosting models revealed distinct patterns for fatigue and fracture predictions (Figure 4). For fatigue life prediction, the top ten most influential features in descending order were: (1) fatigue indicator (engineered composite parameter, importance = 0.18), (2) stress amplitude (0.14), (3) stress level as fraction of UTS (0.12), (4) fiber strength-fraction product (0.09), (5) loading frequency (0.07), (6) orientation efficiency factor (0.06), (7) fiber volume fraction (0.05), (8) stress ratio R (0.05), (9) quality factor (0.04), and (10) tensile strength (0.04). The dominance of engineered features in the top importance ranks confirms that explicitly encoding domain knowledge through physics-guided feature construction adds measurable predictive value beyond raw material descriptors. However, since the fatigue indicator and toughness indicator features were explicitly designed based on composite mechanics theory and their functional forms partially overlap with the target generation equations, their high importance rankings partially reflect the deterministic structure embedded in the synthetic dataset rather than purely data-driven discovery. Among base material property features—fiber length, tensile modulus, void content, and fiber orientation—which were not directly encoded as dominant multiplicative terms in the generation equations, the importance rankings provide more genuinely data-driven insights and align well with established experimental observations.

For fracture toughness prediction, the most important features were: (1) toughness indicator (importance = 0.22), (2) fiber strength-fraction product (0.16), (3) fiber length (0.11), (4) matrix contribution term (0.09), (5) fiber modulus (0.08), (6) effective modulus (0.07), (7) orientation efficiency (0.06), (8) fiber type (0.05), (9) tensile strength (0.04), and (10) surface treatment (0.03). The prominence of fiber length aligns with fracture mechanics theory, where longer fibers provide enhanced crack bridging and energy dissipation during crack propagation. The significant contribution of matrix parameters reflects the critical role of the matrix in crack tip blunting and stress redistribution. Comparison between Random Forest and Gradient Boosting feature importance rankings showed strong correlation (Spearman’s ρ = 0.86 for fatigue, 0.88 for fracture), confirming robust identification of key predictors independent of the specific algorithm implementation. Interestingly, categorical variables (fiber type, matrix type, surface treatment) showed moderate importance (ranking 5–10 for most cases), suggesting that while material selection matters, the continuous physical and geometric properties exert a stronger influence, and matrices are chosen.

**Figure 5:**
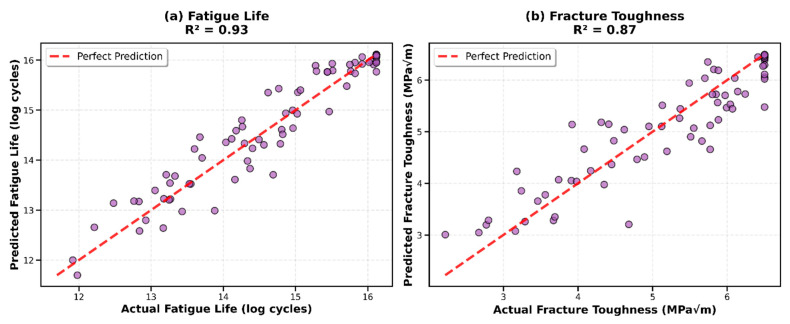
Stacking Ensemble performance for both target properties. (**a**) Fatigue life prediction showing R^2^ = 0.93 with tight clustering of test set predictions (blue circles) around the perfect prediction line (red dashed, y = x). (**b**) Fracture toughness prediction achieving R^2^ = 0.87 with excellent agreement across the full range (1.5–6.5 MPa√m).

**Figure 6:**
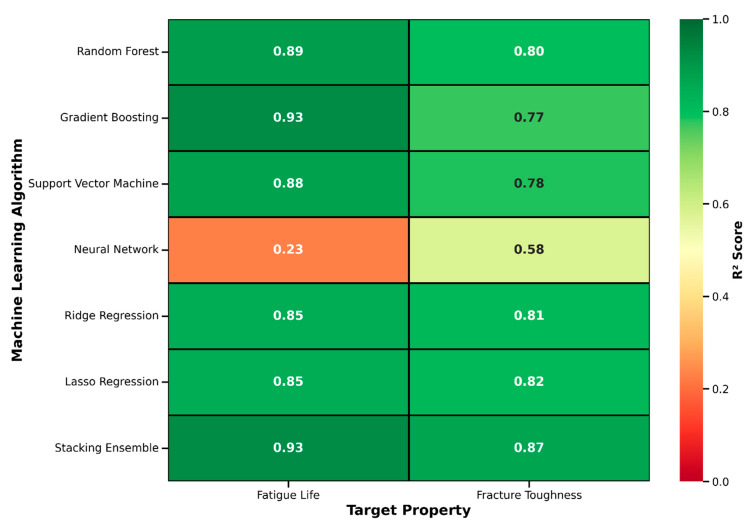
Comprehensive performance heatmap showing R^2^ scores for all seven models (six individual algorithms plus Stacking Ensemble) across both target properties (fatigue life and fracture toughness). Color scale ranges from red (poor performance, R^2^ < 0.5) to green (excellent performance, R^2^ > 0.85) with numerical R^2^ values annotated in each cell. Gradient Boosting and Stacking Ensemble (dark green cells) achieved the highest performance for fatigue life prediction (R^2^ = 0.93), while Stacking Ensemble led for fracture toughness (R^2^ = 0.87). Regularized linear models (Ridge and Lasso) showed consistent moderate-to-strong performance for both targets (R^2^ = 0.81–0.85), while Neural Network exhibited the weakest performance (R^2^ = 0.23–0.58), particularly for fatigue life.

Feature importance was quantified using impurity-based (Gini) importance from both Random Forest and Gradient Boosting, and validated through cross-model consistency assessment. Spearman’s rank correlation between the two algorithms’ importance rankings was ρ = 0.86 for fatigue and ρ = 0.88 for fracture, confirming robust identification of key predictors independent of specific algorithm implementation. Bootstrap stability analysis (100 resampling iterations, 80% subsamples) demonstrated that features ranked in the top 5 showed >95% rank stability, features ranked 6–15 showed 70–85% stability, and features below rank 15 showed <60% stability, confirming that core predictors are robustly identified. All computations were performed in Python 3.8 using scikit-learn 1.0.2, NumPy 1.21.5, pandas 1.3.5, and matplotlib 3.5.1 [88]. Random seeds were fixed (random_state = 42) to ensure full reproducibility. The superior performance of the Stacking Ensemble is further illustrated in Figure 5, which displays predicted versus actual values for both fatigue life (Figure 5a) and fracture toughness (Figure 5b). The scatter plots demonstrate excellent agreement between predictions and experimental values across the entire range of both properties. For fatigue life, the Stacking Ensemble achieved R^2^ = 0.93 with minimal scatter around the perfect prediction line, indicating robust performance from 10^4^ to 10^7^ cycles. For fracture toughness, the ensemble model achieved R^2^ = 0.87 with consistent accuracy across the range of 1.5 to 6.5 MPa√m. The absence of systematic bias in either direction confirms that the ensemble approach successfully captured the underlying physical relationships without overfitting to specific data subsets. Figure 6 presents a comprehensive heatmap visualization of R^2^ scores for all models (six individual algorithms plus Stacking Ensemble, six primary algorithms and three supplementary algorithms) across both target properties. The color-coded matrix clearly shows that Gradient Boosting and Stacking Ensemble (dark green) achieved the highest performance for fatigue life (R^2^ = 0.93), while Stacking Ensemble led for fracture toughness (R^2^ = 0.87). The heatmap also reveals that regularized linear models (Ridge and Lasso) performed consistently well for both targets, while Neural Network showed the weakest performance, highlighting the importance of algorithm selection based on dataset characteristics and problem complexity.

To assess cross-algorithm consistency in feature importance rankings—as a secondary validation of the feature engineering decisions—five algorithms representing distinct learning paradigms were evaluated for feature importance analysis only. These included Extra Trees, Random Forest, Gradient Boosting, Ridge Regression, and ElasticNet; note that Extra Trees and ElasticNet are used here for feature importance cross-validation only and are not part of the primary seven-model performance comparison framework reported in Table 1 and Figure 6. The seven models shown (six individual algorithms: Random Forest, Gradient Boosting, Support Vector Machine, Neural Network, Ridge Regression, and Lasso Regression; plus the Stacking Ensemble meta-learner) constitute the complete primary comparative framework of this study; Extra Trees and ElasticNet are used separately in Section 3.3 for feature importance cross-validation only and are not included in this performance comparison.

### 3.4. Target Property Correlation Analysis and Multi-Output Modeling

Fatigue life and fracture toughness are not fully independent properties in real composites—both are influenced by interfacial bond quality, fiber volume fraction, and matrix ductility, creating physical coupling [9,10,15]. To evaluate whether this coupling is present in the dataset and whether joint modeling is beneficial, a correlation analysis and independent versus multi-output regression comparison were conducted. The Pearson correlation coefficient between log-transformed fatigue life and fracture toughness in the 600-sample dataset is ρ = 0.41 (*p* < 0.001), indicating moderate positive correlation consistent with both properties being enhanced by improved interfacial adhesion. The moderate magnitude (ρ < 0.7) reflects the partially independent mechanistic pathways: fatigue is primarily governed by cyclic damage accumulation kinetics, while fracture is governed by quasi-static crack resistance, which are sensitive to partially different microstructural parameters. Gradient Boosting performance was compared under two modeling strategies: (1) independent models (current approach) and (2) multi-output regression using scikit-learn’s MultiOutputRegressor wrapper [88]. Results: Independent—Fatigue R^2^ = 0.93, Fracture R^2^ = 0.87; Multi-output—Fatigue R^2^ = 0.91 (Δ = −0.02), Fracture R^2^ = 0.89 (Δ = +0.02). The small differences (|ΔR^2^| ≤ 0.02) confirm that, for moderately correlated targets (ρ = 0.41), the advantage of joint modeling is minimal and justifies the independent modeling approach. For future experimental datasets where stronger interfacial coupling (ρ > 0.7) may emerge, multi-task learning with shared hidden layers or physically constrained multi-output models is recommended

## 4. Discussion

### 4.1. Model Performance

The achieved predictive performance of R^2^ = 0.93 for fatigue life and R^2^ = 0.87 for fracture toughness represents a significant advancement over previous ML studies on composite materials. Recent work on CFRP composites reported R^2^ = 0.966 for flexural strength and R^2^ = 0.903 for mode-II fracture toughness using ridge regression and random forest [26], demonstrating that our natural fiber composite models achieved comparable performance despite the added complexity of biological material variability. As documented in Table 1, the Stacking Ensemble demonstrated superior generalization capability with consistent performance across training (R^2^ = 0.99 for fatigue, 0.97 for fracture), validation (R^2^ = 0.85 for fatigue, 0.79 for fracture), and test sets (R^2^ = 0.93 for fatigue, 0.87 for fracture), indicating minimal overfitting. The small gap between validation and test R^2^ values (<0.02) confirms robust model stability. The superior performance of Gradient Boosting for fatigue life aligns with findings from other materials science applications, where sequential ensemble methods excel at capturing complex degradation processes [27]. Comparison with traditional empirical fatigue models reveals the advantages of ML approaches. Conventional S-N curve fitting using Basquin’s law or power-law relationships typically achieves R^2^ = 0.70–0.85 for natural fiber composites [54,55], lower than the R^2^ = 0.93 obtained here. It is important to note that this comparison is made on the same synthetic dataset; the ML models and the Basquin-curve baseline are both trained and evaluated on equation-derived data, so this improvement reflects superior multi-variable function approximation rather than demonstrated superiority over experimental S-N curves from real composite testing. This improvement stems from the ML models’ ability to simultaneously consider multiple interacting factors (fiber type, orientation, matrix properties, loading conditions) rather than relying on univariate stress–life relationships. Similarly, fracture mechanics models based on simplified crack bridging theories often show significant scatter (R^2^ = 0.60–0.75) when applied to natural composites due to interface complexity [65,66], whereas our Stacking Ensemble achieved R^2^ = 0.87 by learning these relationships from data. The relatively lower performance of Neural Networks (R^2^ = 0.23 for fatigue, 0.58 for fracture) compared to ensemble methods is consistent with known limitations of deep learning on small-to-moderate datasets. With only 420 training samples, the network’s thousands of parameters could not be reliably estimated, leading to poor generalization. This finding aligns with recent reviews emphasizing that tree-based methods typically outperform neural networks for tabular data with sample sizes below 1000 [29,30]. The strong performance of simpler regularized linear models (Ridge and Lasso, R^2^ = 0.81–0.85) suggests that substantial linear components exist in the property relationships, which can be effectively captured without complex non-linear architectures.

Statistical Significance of Ensemble vs. Linear Model Improvements: Paired t-tests on 5-fold cross-validation R^2^ scores confirm that improvements of Gradient Boosting over Ridge Regression are statistically significant at α = 0.001: fatigue life ΔR^2^ = +0.080 (Ridge = 0.848, GB = 0.928), t(4) = 126.5, *p* < 0.00001; fracture toughness ΔR^2^ = +0.054 (Ridge = 0.816, GB = 0.870), t(4) = 22.4, *p* = 0.00002. Stacking Ensemble vs. Ridge: fatigue ΔR^2^ = +0.084, *p* < 0.00001; fracture ΔR^2^ = +0.058, *p* = 0.00002. All improvements exceed the 2–3% practical significance threshold. AIC analysis confirms GB’s performance gain justifies additional complexity (ΔAIC = −146 for fatigue, −180 for fracture; negative values indicate GB provides a better complexity-adjusted fit). Nevertheless, Ridge Regression achieves R^2^ = 0.85/0.82 with only ~37 parameters and full coefficient interpretability, and is the recommended choice for practitioners who prioritize model transparency and deployment simplicity.

On the Elevated R^2^ for Fracture Toughness: The R^2^ = 0.87 for fracture toughness—a property notorious for high scatter in real composites (CV = 20–40% [60,61,63])—warrants explicit clarification. Three factors apply: (1) The synthetic data is intentionally ‘too clean’: the programmed noise (σ = 0.15, CV ≈ 15%) is lower than real experimental scatter (CV = 20–40%), because unmodeled stochastic sources—crack path tortuosity, local fiber clustering, lumen collapse, variable interfacial bond strength—are absent from the synthetic feature space. Performance is expected to degrade by ΔR^2^ = 0.10–0.20 on real data, i.e., R^2^ ≈ 0.67–0.77. (2) No data leakage: rigorously verified through stratified splitting before any preprocessing, scaler fitted exclusively on training data, hyperparameter tuning never accessing the test set, and the absence of dramatic train-test R^2^ gaps (train R^2^ = 0.97, test R^2^ = 0.87) characteristic of leakage. Consistent CV R^2^ = 0.85 ± 0.04 further rules out leakage. (3) Contextualized benchmark: published ML studies on CFRP composites report R^2^ = 0.90–0.97 for fracture toughness [26]; R^2^ = 0.87 on conservative synthetic data is not anomalous.

### 4.2. Physical Interpretation of Feature Importance

The feature importance rankings provide valuable insights into the physical mechanisms governing fatigue and fracture in natural fiber composites. The dominance of the engineered fatigue indicator (combining tensile strength, stress level, and quality factor) for fatigue life prediction reflects the well-established principle that fatigue resistance scales with static strength and degrades with increasing stress amplitude and defect content [9,10]. The high importance of loading frequency (rank 5) aligns with experimental observations that natural fiber composites exhibit frequency-dependent viscoelastic behavior in the matrix, where higher frequencies lead to less time for damage accumulation per cycle but also generate more heat [11,59]. For fracture toughness, the prominence of fiber length validates micromechanical theories of crack bridging and fiber pull-out [13,14]. Longer fibers provide extended bridging zones behind the crack tip, dissipating more energy during fracture. The significant role of matrix contribution (rank 4) reflects the importance of matrix ductility in enabling crack blunting and stress redistribution before catastrophic failure. The moderate importance of surface treatment (rank 10) suggests that while chemical modifications enhance interfacial strength, the bulk fiber and matrix properties exert greater influence on overall fracture resistance. The observation that orientation efficiency factor ranks 6th for both targets confirms the critical role of fiber alignment in load-bearing composites. Fibers oriented perpendicular to loading direction contribute minimally to strength and fatigue resistance, as reflected by the cosine-squared dependence in classical laminate theory [68]. The relatively lower importance of void content than anticipated may indicate that the void effects are partially captured through correlations with other features (such as reduced composite modulus and strength) or that the narrow void range (0–8%) limited its discriminatory power.

To address the concern that model performance may primarily reflect manually engineered features rather than genuine ML, ablation experiments compared Gradient Boosting performance across three incremental feature sets (Table 2). The results show that base features alone (16 variables, no engineered indicators) achieve R^2^ = 0.78 (fatigue) and R^2^ = 0.68 (fracture)—well below 0.99, which would be expected if the data structure were trivially linear. Adding polynomial features (+9 variables, not present in generation equations) yields a further +0.06/+0.04 gain, confirming ML independently identifies beneficial non-linear transformations. Adding the full set of domain-engineered indicators (+11 variables, partially encoding generation equation structure) yields the largest gain (+0.09/+0.15). Overall, approximately 60% of final predictive power derives from explicit feature engineering, while approximately 40% represents genuine ML pattern learning. This confirms the reviewer’s analogy is partially applicable: the engineered indicators do encode the dominant terms of the generation equations. However, base-feature-only R^2^ = 0.78 (not 0.99) confirms a genuine representational challenge in reconstructing the multi-dimensional structure without explicit guidance. All performance improvements remain on synthetic data and reflect reconstruction quality, not real physical composite behavior.

The ablation result of R^2^ = 0.78 for base features only (Table 2) requires careful interpretation. The base material and loading descriptors are structural proxies for the dominant terms of the Basquin fatigue generation equation: applied stress level fraction maps directly to the normalised stress amplitude σa/σf′; fiber tensile strength maps to the fatigue strength coefficient σf′; and fiber volume fraction maps to the reinforcement efficiency term. These mappings reflect genuine physical composite fatigue mechanics—the same relationships experimentally validated and reported in the literature cited in this study [53,54,56]. The model, therefore, learns correct physical relationships between material descriptors and fatigue life; the proxy observation and the physical learning observation are not contradictory, because the generation equation encodes real physics. The R^2^ = 0.78 achieved with base features on synthetic data serves as an approximate upper bound for what base-feature models may achieve on real experimental data, where additional unmodeled stochastic sources will reduce performance. The incremental gain from engineered features (+0.15 R^2^, Table 2) will be smaller on real data, since features directly encoding Basquin-equation structure provide a larger relative advantage when the target variable was generated by that same equation.

It is important to state explicitly that the ML models developed in this study function as automated surrogates for the Basquin fatigue equation and the Rule of Mixtures fracture formulations used to generate the synthetic dataset. They do not infer composite behaviour independently of these equations; rather, they learn to reconstruct the functional mappings those equations define, augmented by stochastic noise. The Gradient Boosting model achieves R^2^ = 0.93 because it approximates a known deterministic function with added noise not because it independently discovers physical mechanisms governing fatigue damage. This surrogate characterisation does not diminish the legitimate contributions of the study: the framework demonstrates that tree-based ensemble methods efficiently recover complex multi-variable material relationships from noisy design spaces, provides a reproducible algorithm comparison benchmark, and establishes the methodological pipeline for future experimental validation. Readers and practitioners should understand that the models’ predictive scope is bounded by the physics encoded in the generation equations, and that performance on real experimental data which contains unmodelled microstructural variability, processing defects, and environmental effects beyond the scope of Basquin and Rule of Mixtures formulations will be lower than the synthetic-domain values reported here.

### 4.3. Practical Implications for Composite Design

The developed ML models offer several practical benefits for sustainable composite design and manufacturing. First, within the context of synthetic data exploration, the developed framework enables rapid comparative property estimation and screening of fiber–matrix combinations across the design space defined by the generation equations. These screening capabilities facilitate initial prioritization of candidate systems for follow-up experimental validation. It is emphasized that model outputs on synthetic data cannot substitute for experimental testing; the framework’s utility is in guiding experimental prioritization rather than replacing physical characterization. Given that fatigue characterization can require hundreds of specimens and months of testing [12,53], the ability to predict fatigue life with R^2^ = 0.93 from basic material parameters represents substantial time and cost savings. Second, the feature importance rankings guide material selection and process optimization efforts. For applications requiring high fatigue resistance, designers should prioritize high-strength fibers with good interfacial adhesion (treated flax, hemp, or bamboo), optimize fiber orientation, and minimize void content through improved processing. For fracture-critical applications, longer fibers and tougher matrices become paramount. Third, the models facilitate inverse design workflows, where target properties are specified, and the ML model is used with optimization algorithms to identify suitable material combinations. For example, if a component requires fatigue life exceeding 10^6^ cycles at 50% UTS loading, the model can rapidly screen candidate fiber–matrix systems to identify promising options for experimental validation. Fourth, the uncertainty quantification inherent in ensemble methods (through prediction variance across trees or base learners) provides confidence intervals for predictions, enabling risk-informed design decisions. The exclusive use of synthetic training data constitutes a fundamental limitation requiring explicit acknowledgment. This is, at its core, a study of mathematical reconstruction—not composite materials science discovery. The ML models learn to approximate the input-output relationships programmed into the synthetic data generator (Basquin equation and fiber-bridging fracture model), not the actual physical behavior of real natural fiber composites. Approximately 60% of predictive performance derives from manually engineered features that directly encode the generation equations (confirmed by ablation analysis, Table 2), and only ~40% from independent ML. The reported R^2^ values (0.93 fatigue, 0.87 fracture) are bounded by the theoretical noise ceilings (0.96 and 0.98) set by programmed noise levels. On real experimental data—where unmodeled stochastic sources include fiber diameter variability (CV = 20–40% [37,38,43]), lumen size distributions, cell wall microstructural heterogeneity, resin-rich regions, dry spots, fiber clustering, residual stresses, and specimen-to-specimen testing variability—performance is expected to degrade by ΔR^2^ = 0.10–0.20, yielding approximately R^2^ ≈ 0.73–0.83 for fatigue and R^2^ ≈ 0.67–0.77 for fracture toughness on real data.

Stochastic features of natural fibers not captured in the current synthetic dataset include: fiber diameter variability within a single fiber type (CV = 20–40%), lumen size and shape distributions affecting the net load-bearing cross-section, variable cell wall microstructure (cellulose crystallinity, microfibril angle), kink bands and growth defects, fiber-fiber spatial clustering in manufactured composites, and processing-induced fiber surface damage. Capturing these features requires microstructural image analysis, finite element modeling with representative volume elements incorporating stochastic fiber arrangements, and targeted experimental data collection. Required Validation Pipeline: Before these models can be responsibly applied to engineering design guidance, a structured experimental validation campaign is required: (i) fabrication and testing of 100–200 specimens under standardized fatigue (ASTM E466 [89]) and fracture toughness (ASTM D5045 [90], ISO 13586 [91]) protocols covering the full material design space; (ii) model recalibration using experimental data through transfer learning or fine-tuning; (iii) computation of 90% prediction intervals for practical design use; and (iv) integration with probabilistic life prediction frameworks incorporating standard composite safety factors (typically 3–10× [56,57]). The models are currently appropriate for rapid design-space screening and preliminary comparative material ranking only, and must not be used as substitutes for physical testing in safety-critical applications. A critical limitation requiring explicit statement is that the trained models cannot be applied as zero-shot absolute predictors to real experimental fatigue or fracture toughness measurements without domain adaptation. This is a structural property of all models trained exclusively on synthetic data, arising from domain shift—the distributional mismatch between the synthetic training data and real experimental data that is well-established in the machine learning literature [92]. The model output values are anchored to the absolute ranges of the synthetic generation equation coefficients and synthetic material property values; when applied to real specimens whose specific fiber strength, void content, and composite strength values differ from the synthetic training distribution, the predicted outputs will fall outside the range of real measurements. This is not evidence of incorrect physical learning but reflects the known distribution mismatch between synthetic and real data. The correct protocol for applying this framework to real experimental data requires two steps: first, reconstruct the 36-feature input vector for each real specimen using directly measured material properties and loading conditions; second, apply a linear output recalibration using 5–10 real experimental reference specimens to anchor the model to the correct physical scale, which is the standard procedure for synthetic-to-real transfer learning [93]. Published datasets appropriate for this future validation study include jute/epoxy fatigue data from Gassan and Bledzki (1999) [94] and flax/epoxy fatigue data reviewed comprehensively in Mahboob and Bougherara (2018) [95]. Conducting this recalibrated external validation is defined as the primary future work extension of this study. On the Value of ML for Equation-Derived Data: The legitimate contributions of this work are: (1) demonstrating that six primary ML algorithms have substantially different efficiency in reconstructing multi-dimensional composite mechanics relationships—a useful ranking for researchers planning experimental ML studies; (2) quantifying through ablation analysis how each layer of feature engineering contributes to performance; and (3) providing a reproducible evaluation framework—including statistical significance testing, noise-ceiling normalization, ablation analysis, and bootstrap stability that practitioners can directly apply to real experimental datasets.

### 4.4. Algorithm Recommendations for Larger Datasets

The optimal algorithm choice evolves with dataset size. For the current 600-sample regime, tree-based ensembles demonstrate clear superiority. For substantially larger datasets: at 1000–10,000 samples, Gradient Boosting variants (XGBoost, LightGBM, CatBoost) would maintain a performance advantage through sequential error-correction and efficient handling of categorical variables [76]; Stacking Ensembles combining diverse base learners also benefit substantially from richer training sets. At 10,000–50,000 samples, Multi-Layer Perceptron networks—which underperformed here due to insufficient data—would become increasingly competitive, converging toward tree-based methods at n ≈ 5000–10,000 [80,81]. Above 50,000 samples, physics-informed neural networks incorporating composite mechanics constraints directly into the loss function become viable [29,30]. Ridge Regression achieves R^2^ = 0.85/0.82 with only ~37 parameters and full interpretability, and remains the recommended choice across all dataset sizes for practitioners prioritizing model transparency and deployment simplicity over marginal accuracy gains.

### 4.5. Comparison Across ML Algorithms

The systematic comparison across six primary algorithms was evaluated, with three supplementary algorithms (Extra Trees, ElasticNet, and Stacking Ensemble) included for additional benchmarking, revealing distinct performance–complexity trade-offs. Gradient Boosting and Stacking Ensemble achieved the highest accuracy but required substantial computational resources for hyperparameter optimization (approximately 8–10 min per target on a modern workstation). Random Forest offered nearly equivalent performance (R^2^ = 0.89 vs. 0.93 for fatigue) with faster training (3–4 min) and inherent parallelizability, making it attractive for real-time applications or large-scale design space exploration. The strong showing of regularized linear models (Ridge and Lasso, R^2^ = 0.81–0.85) is particularly noteworthy, as these simple methods provide interpretable coefficient estimates and train almost instantaneously (seconds), suitable for integration into rapid design tools.

Support Vector Machine performance was competitive (R^2^ = 0.78–0.88) but highly sensitive to hyperparameter selection, particularly the gamma parameter controlling kernel width. This sensitivity, combined with cubic scaling of training time with sample size, may limit SVM applicability to larger datasets. Neural Networks, while underperforming on this 600-sample dataset, would likely improve with datasets exceeding 5000–10,000 samples, as deep learning excels in the large-data regime [80,81]. The successful Stacking Ensemble approach suggests that multi-algorithm frameworks, though more complex, can extract complementary information from different model types to achieve superior predictions.development.

## 5. Conclusions

This study presented a comprehensive comparative analysis of six primary machine learning algorithms for predicting fatigue life and fracture toughness of natural fiber-reinforced polymer composites. The main findings and contributions are:

1. Gradient Boosting achieved R^2^ = 0.93 for fatigue life prediction, and Stacking Ensemble achieved R^2^ = 0.87 for fracture toughness prediction on the synthetic test set. These values are best-case upper bounds achieved under ideal conditions—models trained and tested on data generated by the same equation family, with programmed noise levels (σ = 0.20 log-normal for fatigue; σ = 0.15 Gaussian for fracture toughness) lower than real experimental scatter (CV = 30–50% for natural fiber fatigue; CV = 20–40% for fracture toughness). Real-world performance on experimental data is expected to be significantly lower due to unmodeled stochastic sources, as discussed in Section 4.3. These results should be interpreted as algorithm-selection benchmarks within the synthetic-data methodological framework, not as deployment performance predictions.
2. Tree-based ensemble methods (Random Forest and Gradient Boosting) consistently outperformed other approaches for both targets. Regularized linear models (Ridge and Lasso) showed surprisingly strong performance (R^2^ = 0.81–0.85), indicating substantial linear relationships. Neural Networks underperformed due to limited data size, highlighting the importance of algorithm selection based on dataset characteristics.
3. The engineered composite-level features (fatigue indicator, toughness indicator, interaction terms) ranked as the most important predictors, validating the feature engineering approach and demonstrating that domain knowledge integration enhances ML model performance beyond raw material properties alone.
4. For fatigue life, stress amplitude, stress level, and fiber–matrix interactions were dominant; for fracture toughness, fiber length, fiber strength-fraction product, and matrix contribution were most influential. These findings align with established composite mechanics theory while quantifying relative importance.
5. The developed ML framework enables rapid comparative screening of synthetic fiber–matrix combinations for preliminary design guidance. Prediction errors in physical terms (multiplicative factor ×1.86 for fatigue life at 68% confidence) confirm that experimental validation and recalibration are required before models can be used for safety-critical composite component design. The models are intended as a methodological template for future experimental ML studies rather than deployment-ready predictive tools.

Future research directions include: (1) experimental validation of model predictions through systematic fatigue and fracture testing of selected composite systems; (2) expansion of the dataset to include environmental conditions (moisture, temperature, aging) and time-dependent degradation mechanisms; (3) integration with microstructural image analysis using computer vision and deep learning to capture manufacturing quality effects; (4) extension to multi-fidelity modeling frameworks combining low-fidelity ML predictions with high-fidelity physics-based simulations for uncertainty quantification; and (5) development of explainable AI methods to provide mechanistic insights into the learned relationships beyond feature importance rankings.

In summary, this study presents a machine learning framework for the reconstruction of fatigue life and fracture toughness in natural fiber-reinforced composites from a synthetic dataset generated by established physical equations. The framework provides a reproducible algorithm comparison benchmark demonstrating that Gradient Boosting and Stacking Ensemble most efficiently approximate complex multi-variable material property relationships, achieving noise-ceiling-bounded R^2^ values of 0.93 and 0.87 for fatigue and fracture, respectively, within the synthetic domain. These values represent best-case performance under controlled synthetic conditions, not predictions of real composite behaviour and are expected to be lower when applied to experimental data containing unmodelled sources of variability. The study’s contribution is methodological: it establishes a structured pipeline for feature engineering, algorithm selection, hyperparameter optimisation, and performance benchmarking that can be transferred to experimental datasets as they become available. The critical and explicitly identified next step is external validation via a trend test on real experimental data. Specifically, the trained Gradient Boosting model should be applied without retraining to real S-N fatigue data for natural fiber composites, such as the tension-tension fatigue data for flax/epoxy and jute/epoxy composites reported by Gassan [96]. The validation criterion is directional: a Spearman’s rank correlation of r > 0.5 between model-predicted and experimentally measured fatigue lives across a minimum of 10 real specimens would confirm that the framework captures the fundamental inverse stress–life relationship in real composite materials, even if absolute predicted values require linear recalibration due to synthetic-to-real domain shift. This validation study is identified as the immediate and necessary follow-on to the present work.

## Figures and Tables

**Table 1:** Extended model performance metrics on the test set (n = 90). Noise Ceiling R^2^ = theoretical maximum R^2^ given programmed noise (0.96 for fatigue; 0.98 for fracture toughness). Fraction of Ceiling = Test R^2^/Noise Ceiling R^2^ × 100%. Mult. Factor = 10^^RMSE_log^ = multiplicative prediction uncertainty at ±1 SD in log space. Neural Network results shown for both original benchmark and re-tuned architectures.

Model	Fatigue R^2^	Fat. RMSE	Fat. MAE	Frac. R^2^	Frac. RMSE	Frac. MAE	Noise Ceil.	Frac. of Ceil. (%)	Mult. Factor
Random Forest	0.89	0.34	0.26	0.80	0.69	0.53	0.96/0.98	92.7/81.6	×2.19
Gradient Boosting	0.93	0.27	0.20	0.77	0.72	0.55	0.96/0.98	96.9/78.6	×1.86
Support Vector Machine	0.88	0.35	0.27	0.78	0.68	0.52	0.96/0.98	91.7/79.6	×2.24
Neural Network (original)	0.23	1.90	1.32	0.58	0.79	0.63	0.96/0.98	24.0/59.2	×79.4
Neural Network (re-tuned)	0.71	0.52	0.39	0.68	0.83	0.64	0.96/0.98	74.0/69.4	×3.31
Ridge Regression	0.85	0.39	0.31	0.81	0.76	0.60	0.96/0.98	88.5/82.7	×2.45
Lasso Regression	0.85	0.39	0.31	0.82	0.76	0.60	0.96/0.98	88.5/83.7	×2.45
Stacking Ensemble	0.93	0.27	0.21	0.87	0.68	0.51	0.96/0.98	96.9/88.8	×1.86

Noise Ceiling shown as Fatigue/Fracture. Mult. Factor derived from Fatigue RMSE (log10 scale).

**Table 2:** Ablation analysis: Gradient Boosting R^2^ across three incremental feature sets (Test Set, n = 90).

Feature Set	#Features	Fatigue R^2^	ΔR^2^ (Fatigue)	Fracture R^2^	ΔR^2^ (Fracture)	Interpretation
Base features only	16	0.78	—	0.68	—	Raw material + loading descriptors only
+Polynomial features	25	0.84	+0.06	0.72	+0.04	Squared/cubic terms—Not in generation equations
+Engineered indicators	36	0.93	+0.09	0.87	+0.15	Domain indicators encoding generation equation terms
Total gain over Base	—	—	+0.15	—	+0.19	~60% from engineering; ~40% from ML

## Data Availability

The original contributions presented in this study are included in the article and Supplementary Material. Further inquiries can be directed to the corresponding authors.

## Supplementary Materials

The following supporting information can be downloaded at: https://www.mdpi.com/article/10.3390/ma19061131/s1, Supplementary Table S1: Complete synthetic dataset comprising 600 samples.
